# Differential inhibition of *Arabidopsis* superoxide dismutases by peroxynitrite-mediated tyrosine nitration

**DOI:** 10.1093/jxb/eru458

**Published:** 2014-11-26

**Authors:** Christian Holzmeister, Frank Gaupels, Arie Geerlof, Hakan Sarioglu, Michael Sattler, Jörg Durner, Christian Lindermayr

**Affiliations:** ^1^Institute of Biochemical Plant Pathology, Helmholtz Zentrum München–German Research Center for Environmental Health, 85764 München/Neuherberg, Germany; ^2^Institute of Structural Biology, Helmholtz Zentrum München–German Research Center for Environmental Health, 85764 München/Neuherberg, Germany; ^3^Department of Protein Science, Helmholtz Zentrum München-German Research Center for Environmental Health, 85764 München/Neuherberg, Germany; ^4^Munich Center for Integrated Protein Science at Chair of Biomolecular NMR, Department Chemie, Technische Universität München, 85747 Garching, Germany; ^5^Chair of Biochemical Plant Pathology, Technische Universität München, 85354 Freising, Germany

**Keywords:** Antioxidant system, nitric oxide, nitrosative stress, post-translational modification, superoxide dismutase, tyrosine nitration.

## Abstract

Despite the importance of superoxide dismutases (SODs) in the plant antioxidant defence system little is known about their regulation by post-translational modifications. Here, we investigated the *in vitro* effects of nitric oxide derivatives on the seven SOD isoforms of *Arabidopsis thaliana*. *S*-nitrosoglutathione, which causes *S*-nitrosylation of cysteine residues, did not influence SOD activities. By contrast, peroxynitrite inhibited the mitochondrial manganese SOD1 (MSD1), peroxisomal copper/zinc SOD3 (CSD3), and chloroplastic iron SOD3 (FSD3), but no other SODs. MSD1 was inhibited by up to 90% but CSD3 and FSD3 only by a maximum of 30%. Down-regulation of these SOD isoforms correlated with tyrosine (Tyr) nitration and both could be prevented by the peroxynitrite scavenger urate. Site-directed mutagenesis revealed that—amongst the 10 Tyr residues present in MSD1—Tyr63 was the main target responsible for nitration and inactivation of the enzyme. Tyr63 is located nearby the active centre at a distance of only 5.26 Å indicating that nitration could affect accessibility of the substrate binding pocket. The corresponding Tyr34 of human manganese SOD is also nitrated, suggesting that this might be an evolutionarily conserved mechanism for regulation of manganese SODs.

## Summary

Superoxide dismutases (SODs) are differentially inhibited by peroxynitrite-mediated tyrosine nitration. Tyr63 is the main target responsible for inactivation of MnSOD1. This mechanism seems to be evolutionarily conserved in multicellular organisms.

## Introduction

In plant cells the reactive oxygen species (ROS) superoxide (O_2_^–^) arises as a potentially harmful by-product of photosynthetic and respiratory electron transport chains. It can also be enzymatically produced by various oxidases to serve as a signal or intermediate in general metabolism, development, and stress responses (Mittler *et al.*, 2011). Independent of origin and function, O_2_^–^ levels are carefully controlled by the antioxidant system (Foyer and Noctor, 2009). O_2_^–^ is either scavenged by antioxidants such as reduced ascorbate and glutathione or is efficiently converted to hydrogen peroxide (H_2_O_2_) by superoxide dismutase (SOD; O_2_^–^ + 2 H^+^→H_2_O_2_ + O_2_). H_2_O_2_ in turn is subsequently degraded to water by catalase and peroxidases. Thus, by controlling O_2_^–^ (and indirectly H_2_O_2_) levels SODs are important regulators of cellular redox homeostasis and signalling.

Plant SODs are commonly classified according to their active site cofactors into manganese SOD (MnSOD), iron SOD (FeSOD), and copper/zinc SOD (CuZnSOD). *Arabidopsis* possesses 7 SOD isoforms namely one MnSOD (MSD1), three FeSODs (FSD1–3), and three CuZnSODs (CSD1–3) (Kliebenstein *et al.*, 1998). Whereas MSD1 has a mitochondrial targeting sequence, FSD2, FSD3, and CSD2 are localized in chloroplasts, CSD1 and FSD1 in the cytosol, and CSD3 in peroxisomes (Huang *et al.*, 2012; Kliebenstein *et al.*, 1998; Myouga *et al.*, 2008). Gene expression of the SOD isoforms is differentially regulated in response to stress treatments known to promote the accumulation of ROS. For instance, ozone fumigation strongly induced CSD1 but repressed CSD3 and FSD1 expression (Kliebenstein *et al.*, 1998). These results suggest that the different SOD isoforms have specific functions under stress conditions. Moreover, SOD transcript levels did not always correlate with protein abundance and enzyme activity indicating that SODs are controlled on multiple levels including post-transcriptional and post-translational mechanisms (Kliebenstein *et al.*, 1998; Madamanchi *et al.*, 1994). In this context it is interesting that recent publications hint at a role of nitric oxide (NO) dependent protein modifications in the regulation of mammalian SODs (Radi, 2013).

NO is an important messenger in many physiological processes (Gaupels *et al.*, 2011*a*; Leitner *et al.*, 2009; Mur *et al.*, 2013; Yun *et al.*, 2011). During stress responses NO often interacts with ROS and antioxidants thereby forming reactive nitrogen species (RNS) (Gross *et al.*, 2013; Hill *et al.*, 2010; Scheler *et al.*, 2013). Such NO derivatives can cause post-translational modifications of proteins by *S*-nitrosylation (^..^NO adduct) of cysteine (Cys) residues and metal groups or nitration (-NO adduct) of tyrosine (Tyr) and tryptophan residues (Arasimowicz-Jelonek and Floryszak-Wieczorek, 2011; Astier and Lindermayr, 2012; Gaupels *et al.*, 2011*a*; Hill *et al.*, 2010; Kovacs and Lindermayr, 2013). *S*-nitrosoglutathione (GSNO), nitrosonium ion (NO^+^), and dinitrogentrioxide (N_2_O_3_) represent major RNS promoting *S*-nitrosylation, whereas peroxynitrite (ONOO^–^) and nitrogen dioxide (NO_2_) mediate protein nitration (Hill *et al.*, 2010). NO-dependent protein modifications have an effect on the activity of antioxidant enzymes. One prominent example is mammalian MnSOD, which can be Tyr nitrated (MacMillan-Crow *et al.*, 1996; Radi, 2013). *In vitro* and *in vivo* under inflammatory conditions MnSOD was site-specifically nitrated at Tyr34, which caused inhibition of SOD activity and consequently disturbance of mitochondrial redox homeostasis (Radi, 2013; Yamakura *et al.*, 1998). Less is known about regulation of plant SODs by NO. Occasionally, SODs of various plant species were listed amongst candidate *S*-nitrosylated and Tyr nitrated proteins (Lin *et al.*, 2012; Sehrawat *et al.*, 2013; Tanou *et al.*, 2009). However, NO-modifications were not confirmed *in vitro* nor was the effect of RNS on SOD activity investigated in any detail.

Here, we report the differential inhibition of *Arabidopsis* SODs by Tyr nitration. We observed that overall SOD activity was decreased in leaf extracts from GSNO-/NO-accumulating GSNO reductase-deficient mutants as compared with WT although the expression of SOD-coding genes was nearly unchanged. From these results we concluded that SOD isoforms might be inhibited by NO-dependent post-translational modifications. This prompted us to undertake a systematic candidate approach for defining the role of RNS in regulation of all seven *Arabidopsis* SOD isoforms. *In vitro* tests demonstrated that SOD activities were not altered upon GSNO treatment but MSD1, FSD3, and CSD3 were inhibited to different degrees by ONOO^–^. Inhibition of the enzymes correlated with increased Tyr nitration. Site-directed mutagenesis revealed that nitration of Tyr63 caused most of the almost complete inactivation of MSD1 by ONOO^–^. In sum, nitration of MSD1 is a good model for post-translational regulation of plant enzymes as a whole and SOD isoforms in particular. Putative physiological effects of SOD inhibition by nitration under stress conditions are discussed.

## Materials and methods

### Plant material

*Arabidopsis thaliana* seeds (ecotype Col-0) were sown on soil:sand mixture (4:1). After vernalization for 2 days (4 °C dark), plants were cultivated in a climate chamber at 60% relative humidity under long-day conditions (16h light/8h dark cycle, 20 °C day/18 °C night regime, 70 µmol m^–2^ s^–1^ photon flux density).

### Cloning and heterologous expression of *Arabidopsis* SODs

For cloning the cDNAs of the different SOD isoforms the lambda phage-based site-specific recombination (Stratagene) was used (Landy, 1989). The isolation of the cDNAs of the different SODs was achieved by RT-PCR using gene-specific oligonucleotides (Supplementary Table S1). Briefly, total RNA extractions were performed from 100mg leaf tissue using the TRIzol reagent according to the supplier’s instructions (Invitrogen). QuantiTect Reverse Transcription Kit (Qiagen) was used to synthesize cDNA according to the protocol of the supplier. The introduction of the DNA recombination sequence (att) at the 5’- and 3’-end of the coding sequence of each isoform was achieved by PCR using the isoform-specific att-primers (Supplementary Table S1) and the amplified cDNAs as template. The resulting PCR products were introduced into pDONR221 by recombination using BP Clonase enzyme mixture according to the instructions of the manufacturer. After verifying the sequences of the different SODs they were transferred into the expression vectors pDEST17 and pDEST42 by recombination using LP Clonase enzyme mixture. pDEST17 and pDEST42 allows production of N-terminal or C-terminal His_6_-tag fusion proteins, respectively. For optimal production different bacterial expression strains were tested (BL21 DE3, Rosetta DE3, and Rosetta DE3 pLysS) and the most productive strain for each SOD was selected.

*E. coli* strains harbouring the different plasmids for production of recombinant SODs were grown in 50ml Luria-Bertani medium at 37 °C overnight. These cultures were used to inoculate 2 l auto-induction medium (Studier, 2005). The bacteria were grown overnight at 37 °C until an OD_600nm_ of 2 was reached. Afterwards bacterial cells were harvested by centrifugation.

### Extraction, purification, and treatments of SODs with GSNO and peroxynitrite

For protein extraction the cells were resuspended in 160ml lysis buffer (50mM Tris-HCl, pH 8.0, 300mM NaCl, 20mM imidazole, 10mM MgCl_2_, 1mM protease-inhibitor AEBSF, 0.02% 1-thioglycerol, 0.2 µg ml^–1^ DNaseI, 1mg ml^–1^ lysozyme) and disrupted by high pressure homogenization and sonification. Cellular debris was removed by centrifugation (25 000g, 1h, 4 °C). The recombinant proteins were purified by affinity chromatography using 1.0ml Ni–NTA agarose in Econo-Pac columns (Biorad, Munich, Germany). The protein extracts were applied onto the columns twice, and washed with 30ml of washing buffer (50mM Tris-HCl, pH 8.0, 300mM NaCl, 20mM imidazole, 0.02% glycerol). Adsorbed proteins were eluted from the matrix in three 5ml fractions with 300mM imidazole in washing buffer. Eluates were frozen in liquid nitrogen and stored at –20 °C until analysis.

The purified enzymes were re-buffered in potassium phosphate buffer (pH 8.0) using Zeba spin columns (Thermo Scientific, Rockford, USA). Afterwards, the enzymes were treated with 250 µM and 500 µM GSNO for 20min (RT, in dark). Control treatment was done with 500 µM GSNO in presence of 5mM DTT. Alternatively, purified SODs were treated for 20min with different concentrations of ONOO^–^ (RT, in dark). ONOO^–^ was purchased from Calbiochem (Darmstadt, Germany) in 4.7% NaOH at 160–200mM. The exact concentration was determined according to the manufacturer′s instructions. Control treatment was done with 500 µM ONOO^–^ in presence of 100 µM urate. Excess GSNO, DTT, ONOO^–^, and urate were removed with Zeba spin columns before determination of SOD activities.

The activity of the purified, recombinant SODs was determined using the nitroblue tetrazolium (NBT)–formazan method (McCord and Fridovich, 1969) or the cytochrome *c*-based assay (McCord, 2001).

### Detection of SOD nitration by anti-nitrotyrosine western blot

Proteins were separated by SDS-PAGE on 12% polyacrylamide gels (Laemmli, 1970), transferred onto PVDF membranes, and blocked with 1% non-fat milk powder and 1% bovine serum albumin. The blots were incubated with goat anti-nitrotyrosine antibody (1:2000) at 4 °C overnight, followed by incubation with rabbit anti-goat IgG conjugated with horseradish peroxidase (1:3000) (Invitrogen, Darmstadt, Germany) for 1h at RT. Cross-reacting protein bands were visualized via chemiluminescence using the West Pico Chemiluminescence Detection Kit (Thermo Scientific, Rockford, USA).

### Site-directed mutagenesis

The modification of single nucleotide residues was performed as previously described (Lindermayr *et al.*, 2003). Briefly, for mutation, a pair of oligonucleotides was synthesized harbouring the desired alterations (Supplementary Table S1). For amplification, 60ng plasmid DNA was used in a total volume of 10 µl, including 1 µM each primer, 200 µM dNTPs, and 1U of iProof DNA polymerase. After denaturation (1min at 98 °C) 20 cycles were conducted, consisting of 25 s at 98 °C, 55 s at 55 °C, and 6min at 72 °C, followed by a final extension step at 72 °C for 10min. Subsequently, the parental and hemi-parental template DNA was digested with *Dpn*I and the amplified plasmids were transformed into *E. coli* DH5α. The mutation was verified by sequencing.

### Modelling of the 3D structure of MSD1

Amino acid sequences were aligned and modelled using SWISS-Model (www.expasy.ch). The crystal structure of *Caenorhabditis elegans* MnSOD (PDBcode: PDB 3DC6) was used as template for the prediction of the putative conformation of *Arabidopsis* MSD1. Pymol software (DeLano Scientific, Portland, USA) was used for model visualization.

### Nano-HPLC-MS^2/3^ and data analysis

For mass spectrometric analyses proteins were digested with trypsin at 37 °C for 16h in 50mM NH_4_HCO_3_, pH 8.0. The used trypsin/protein ratio was 1/20. All nano-HPLC-MS^2/3^-experiments were performed on an Ultimate 3000 HPLC nanoflow system (Dionex) connected to a linear ion trap-Fourier transform mass spectrometer (LTQ-Orbitrap, Thermo Fisher Scientific, San Jose, CA, USA). For LTQ-Orbitrap mass spectrometry, the digested peptides were first separated by reversed-phase chromatography (PepMap, 15cm_75mm id, 3mm/100 Å pore size, LC Packings) operated on a nano-HPLC (Ultimate 3000, Dionex) with a nonlinear 170min gradient using 2% ACN in 0.1% formic acid in water (A) and 0.1% formic acid in 98% ACN (B) as eluents with a flow rate of 250 nl min^–1^. The nano-LC was connected to a linear quadrupole ion trap-Orbitrap (LTQ Orbitrap XL) mass spectrometer (Thermo-Fisher, Bremen, Germany) equipped with a nano-ESI source. The mass spectrometer was operated in the data-dependent mode to automatically switch between Orbitrap-MS and LTQ-MS/MS acquisition. Survey full scan MS spectra (from *m*/*z* 300–1500) were acquired in the Orbitrap with resolution R560 000 at *m*/*z* 400 (after accumulation to a target value of 1 000 000 charges in the LTQ). The method used allowed sequential isolation of the most intense ions, up to ten, depending on signal intensity, for fragmentation on the linear ion trap using collisionally induced dissociation at a target value of 100 000 ions. High-resolution MS scans in the orbitrap and MS/MS scans in the linear ion trap were performed in parallel. Target peptides already selected for MS/MS were dynamically excluded for 30 s. General conditions were as follows: electrospray voltage, 1.25–1.4kV; no sheath and auxiliary gas flow. The following modifications were set to be variable: nitration of Tyr residues.

## Results

### Cloning, heterologous expression, and purification of *Arabidopsis* SODs

SODs are important enzymes of the antioxidant system and several enzyme activities of this system are affected by NO. Mammalian MnSOD, for instance, is a target for Tyr nitration (MacMillan-Crow *et al.*, 1996; Radi, 2013). Under inflammatory conditions human MnSOD is site-specifically nitrated at Tyr34, which results in inhibition of SOD activity and consequently disturbance of mitochondrial redox homeostasis (Radi, 2013; Yamakura *et al.*, 1998). Less is known about regulation of plant SODs by NO, although SODs of various plant species were identified as candidates for *S*-nitrosylation and Tyr nitration (Lin *et al.*, 2012; Sehrawat *et al.*, 2013; Tanou *et al.*, 2009), NO-dependent modifications were not confirmed until now. In *Arabidopsis* seven different SODs are described, including one MSD, three FSDs, and three CSDs. The deduced amino acid sequences of the different isoenzymes show very different homology among each other (44–46% within the FSDs, 45–57% within the CSDs) (Table 1). Moreover, the identity of the amino acid sequences between MSD1 and FSDs is higher (29–31%) than the identity between MSD1 and CSDs (18–21%), suggesting that MSD1 is closer related to FSDs (Table 1). The corresponding amino acid sequence alignments are provided in the Supplementary data (Figs S1–S4).

**Table 1.** Amino acid sequence identity and similarity between the different Arabidopsis SOD isoforms

	AA sequence identity (%)	AA sequence similarity (%)
FSD1–FSD2	46	57
FSD1–FSD3	44	58
FSD2–FSD3	45	59
CSD1–CSD2	47	53
CSD1–CSD3	57	67
CSD2–CSD3	45	54
MSD1–FSD1	47	53
MSD1–FSD2	57	67
MSD1–FSD3	45	54
MSD1–CSD1	19	28
MSD1–CSD2	21	30
MSD1–CSD3	18	31

**Fig. 1.**
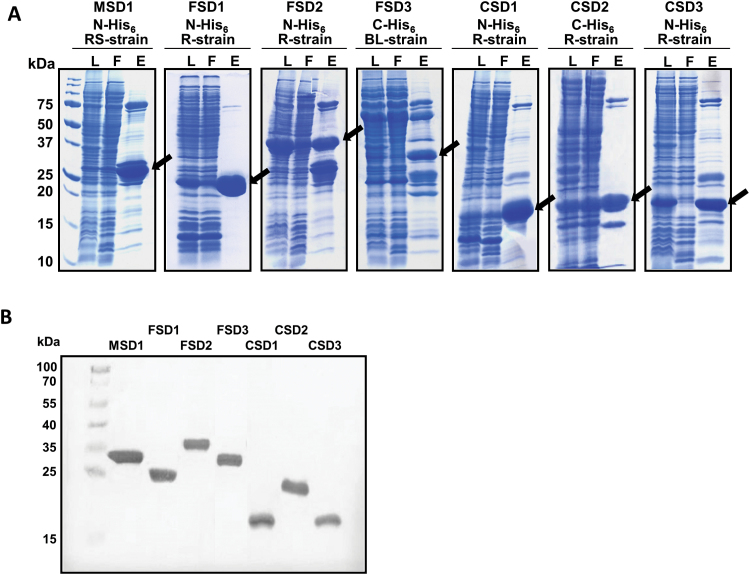
Production, purification, and detection of recombinant *Arabidopsis* SODs. (A) The coding sequences of the different *Arabidopsis* SODs were cloned into pDEST17 (N-terminal His_6_) or pDEST42 (C-terminal His_6_) using the Gateway Technology. Three different bacteria production strains (RS-strain=Rosetta DE3 pLysS; R-strain=Rosetta DE3; BL-strain=BL21 DE3) were tested and the most productive one for each isoform was used. His-tagged SODs were purified by Ni–NTA affinity chromatography. Crude bacterial lysate (L), flow-through (F), and eluate (E) were separated by SDS-PAGE and visualized by Coomassie Blue staining. Arrows indicate the produced SOD isoforms. The relative mass of protein standards are shown on the left. (B) Detection of purified, recombinant *Arabidopsis* SOD isoforms. Eluates containing recombinant SOD isoforms were separated by SDS-PAGE and blotted onto nitrocellulose membrane. Detection of His-tagged proteins was achieved using anti-His antibody. The relative mass of protein standards are given on the left. (This figure is available in colour at *JXB* online.)

We heterologously produced and purified all seven *Arabidopsis* SOD proteins for *in vitro* analyses of their regulation by *S*-nitrosylation of cysteine residues or nitration of Tyr residues. First, we isolated the coding sequence of all seven *Arabidopsis* SOD proteins. The isolation of the cDNAs of the different SODs was achieved by RT-PCR using gene-specific oligonucleotides and the amplified coding sequences were expressed in *Escherichia coli* as fusion proteins containing either N-terminal or C-terminal His_6_-tags. For optimal production different bacterial expression strains were tested (BL21 DE3, Rosetta DE3, and Rosetta DE3 pLysS) and the most productive strain for each SOD was selected. After affinity chromatography on Ni–NTA–agarose, the seven proteins showed the expected relative molecular masses in SDS-polyacrylamide gels and on the immunoblot (Fig. 1).

**Fig. 2.**
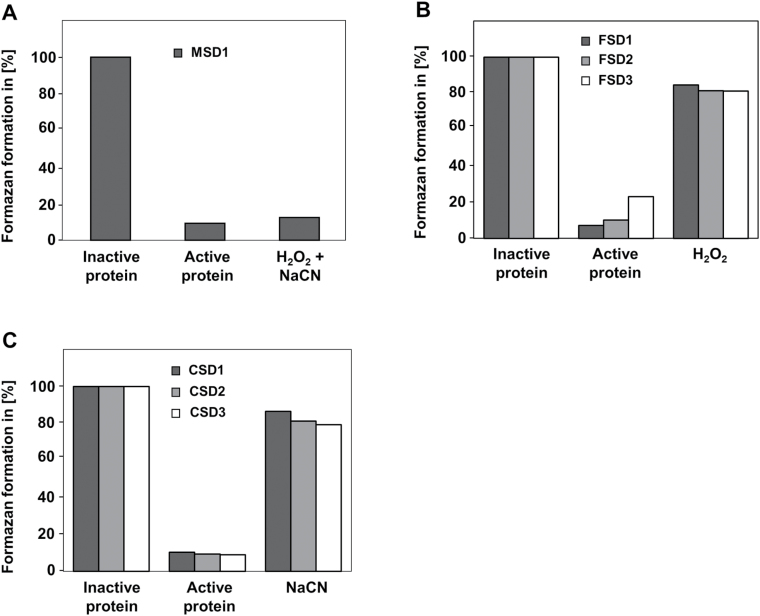
Enzyme activities of purified, recombinant SODs. Shown is the inhibition of formazan formation by MSD1 (A), FSDs (B), and CSDs (C). Formazan formation with heat-inactivated protein extracts was set to 100%. To distinguish between the different SOD types specific inhibitors (H_2_O_2_ for FSDs and NaCN for CSDs) were used. MSD1 is insensitive to both inhibitors.

The activity of the purified, recombinant SODs was determined using the nitroblue tetrazolium (NBT)–formazan method (Fig. 2). In this assay, O_2_^–^ ions are generated from the conversion of xanthine and O_2_ to uric acid and H_2_O_2_ by xanthine oxidase. The O_2_^–^ anion then converts a NBT into a formazan dye. Addition of SOD to this reaction reduces O_2_^–^ ion levels, thereby lowering the rate of formazan dye formation. SOD activity is monitored at a wavelength of 570nm and determined as the percent inhibition of the rate of formazan dye formation. The different types of SODs were verified using specific inhibitors (H_2_O_2_ for FSDs and NaCN for CSDs). MSD1 is insensitive to both inhibitors (Fig. 2).

### MSD1, FSD3, and CSD3 are inhibited by ONOO^–^

**Fig. 3.**
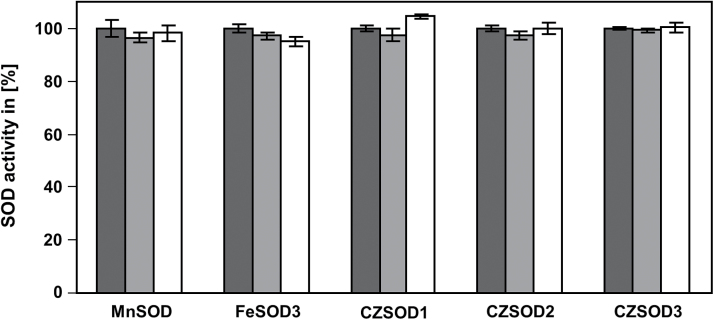
Effect of GSNO on enzyme activity of cysteine containing SODs. Recombinant MnSOD, FeSOD3, Cu/ZnSOD1, Cu/ZnSOD2, and Cu/ZnSOD3 were treated with 250 µM (light grey) and 500 µM (white) GSNO for 20min (RT, in dark). Control treatment was done with 500 µM GSNO in presence of 5mM DTT (dark grey). Afterwards the activity was determined. Treatment with light-inactivated GSNO was used as control. These activities were set to 100%. Values represent means±SD of three independent experiments.

**Fig. 4.**
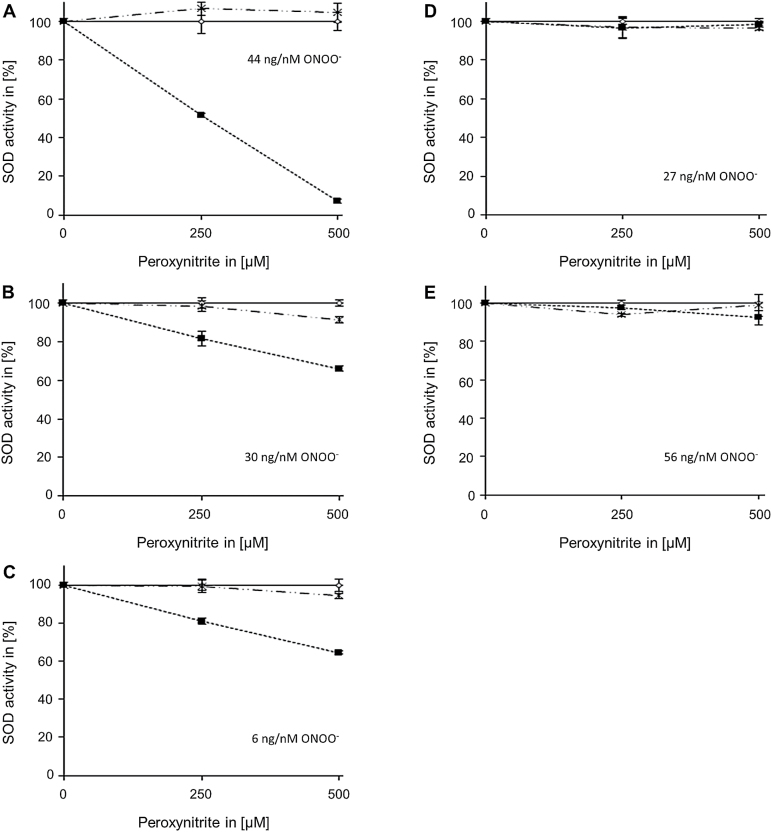
Effect of peroxynitrite on enzyme activity of Tyr-containing SODs. Recombinant MSD1 (A, 22 µg), FSD3 (B, 15 µg), Cu/ZnSOD3 (C, 3 µg), FSD1 (D, 13 µg), and FSD2 (E, 28 µg) were treated with peroxynitrite for 20min (RT, in dark). Afterwards the activity was determined by monitoring reduction of cytochrome *c*. The given values indicate the ratio of applied protein per nmol ONOO^–^ calculated for the highest ONOO^–^ used (500 µM). Filled squares: peroxynitrite treatment; open squares: peroxynitrite treatment in presence of 100 µM urate; crosses: treatment with decomposed peroxynitrite. The activities of urate-treated samples were set to 100%. Values represent means±SD of three independent experiments.

The total SOD activity in *atgsnor* plants is lower than in WT plants (Supplementary Fig. S5), which is probably related to the higher levels of NO-derivatives in the mutant (Feechan *et al.*, 2005). As the decreased SOD activity in *atgsnor* cannot be explained by transcriptional regulation (Supplementary Fig. S6), we hypothesized that it is regulated on the protein level. The two most important NO-dependent post-translational modifications are *S*-nitrosylation of Cys residues and nitration of Tyr residues. Assuming that SOD activity might be inhibited by *S*-nitrosylation of critical Cys residues, MSD1, FSD3, and all three CSDs, were treated with the *S*-nitrosylating agent GSNO, as these isoform have at least one cysteine residue. However, none of these SODs was inhibited by GSNO (Fig. 3). Next, we tested the effect of ONOO^–^ on SOD activity. To this end, all SODs, which have at least one Tyr residue (MSD1, all three FSDs, and CSD3) were treated with different concentrations of ONOO^–^. A concentration-dependent inhibition of MSD1, FSD3, and CSD3 could be observed, whereas the activity of the other two tested FSD isoforms was not affected by this treatment (Fig. 4). Especially MSD1 seems to be very sensitive to this treatment. Its activity decreased to about 10% with 500 µM ONOO^–^, whereas the activity of FSD3 and CSD3 was reduced to 65%. However, it has to be mentioned that the observed differences in the efficiency of ONOO^–^-dependent inhibition of the different SODs could be caused by different ratio of applied protein and ONOO^–^. For a better comparison we calculated the ratio of applied protein per nmol ONOO^–^ for the highest ONOO^–^ concentration used (500 µM) (Fig. 4).

**Fig. 5.**
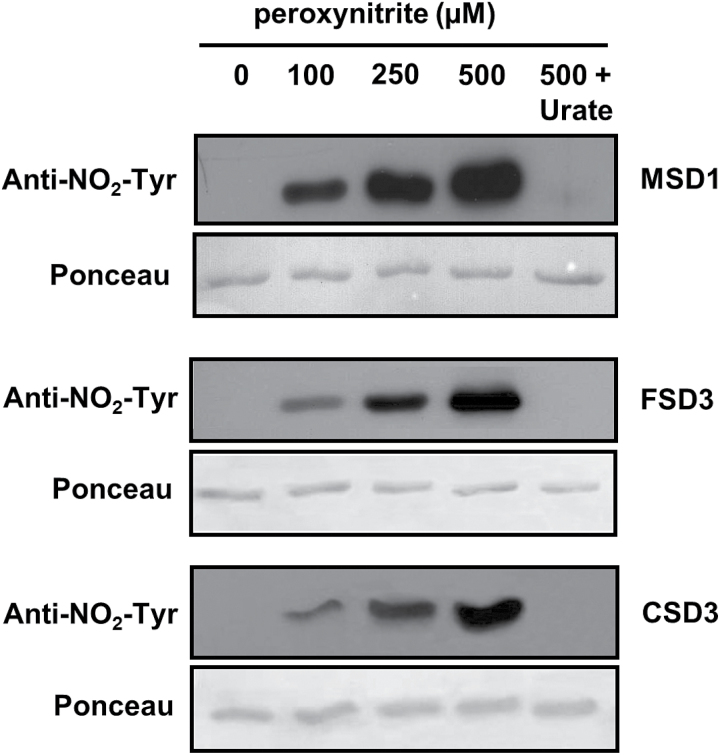
Detection of nitrated Tyr residues. Purified, recombinant MSD1, FSD3, and Cu/ZnSOD3 were treated with different concentrations of peroxynitrite, separated by SDS-PAGE, and blotted onto nitrocellulose membrane. Detection of nitrated Tyr residues was achieved using anti-NO_2_–Tyr antibody. Treatment with 500 µM peroxynitrite in presence of 100 µM urate was used as control.

Inhibition of enzyme activity by ONOO^–^ correlated with increased protein nitration as detected by immunoblot analyses using an anti-nitrotyrosine antibody (Fig. 5). Notably, western blot signals were stronger for MSD1 than FSD3 and CSD3. Because of the high sensitivity of MSD1 to ONOO^–^ this isoform has been analysed in more detail.

### Mass spectrometric identification of nitrated Tyr residues in MSD1

**Fig. 6.**
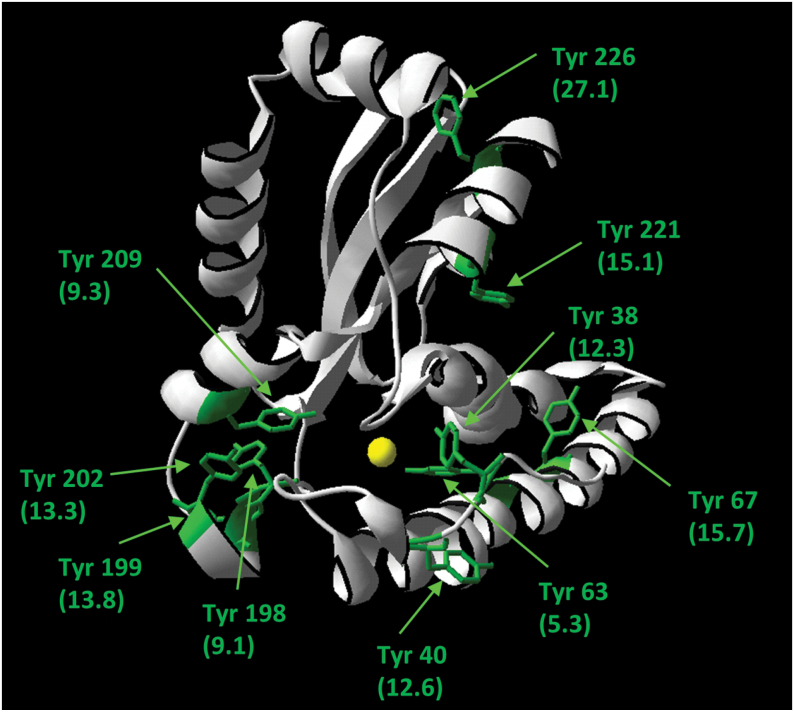
Structural model of *Arabidopsis* MSD1. The structural model of *Arabidopsis* MSD1 was generated using SWISS-MODEL with the crystal structure of *Caenorhabditis elegans* MnSOD as template (PDBcode: PDB 3DC6). The Tyr residues are marked in green. The distances between Tyr side chains and the active side manganese ion (yellow) is given in Ångström in brackets.

To identify the modified Tyr residues in MSD1, peroxynitrite-treated MSD1 was analysed by mass spectrometry. In total, MSD1 has ten Tyr residues. Modelling of the three-dimensional structure of MSD11 revealed that especially Tyr63, Tyr198, and Tyr209 were located close to an active site manganese ion at a distance lower than 10 Å (5.3 Å, 9.1 Å, 9.3 Å, respectively) (Fig. 6). MSD1 was treated with 500 µM peroxynitrite and digested with trypsin. This protease generated analysable peptides containing the different Tyr residues mentioned above. For each nitrated Tyr residue an increase in mass by 45Da was expected. All identified nitrated Tyr residues are summarized in Table 2. Tyr residues 209, 221, and 226 are not accessible to nitration, as they were only found in their unmodified form. Especially nitration of Tyr63, which is closest to the active site manganese, could be of special importance for the inhibitory effect of peroxynitrite on MSD1, as it corresponds to Tyr34 in human MnSOD.

**Table 2.** Determination of Tyr nitration of MSD1 by mass spectrometryPurified, reduced, recombinant MSD1 was incubated with 500 µM peroxynitrite and digested with trypsin. Peptides containing at least one Tyr residue were analysed by mass spectrometry to determine Tyr nitration. Expected (single charged) and observed (multiple charged) *m*/*z* values for the different peptides are shown.

**Identified peptide**	**Mascot**	***m*/*z***	***m*/*z***	**charge**	**modification**
	**Score**	**(expected)**	**(observed)**		
KHHQAYVTNY^67^NNALEQLDQAVNKG	76	1.307	1.308	2	Nitro (+45)
KHHQAY^63^VTNYNNALEQLDQAVNKGDASTVVKL	70	0.843	0.844	4	Nitro (+45)
KGGSLVPLVGIDVWEHAY^198^YLQYKN	46	1.276	1.277	2	Nitro (+45)
KGGSLVPLVGIDVWEHAYY^199^LQYKN	45	1.276	1.277	2	Nitro (+45)
KGGSLVPLVGIDVWEHAYYLQY^202^KN	42	1.276	1.277	2	Nitro (+45)
RGIQTFTLPDLPYDY^40^GALEPAISGEIMQIHHQKH	39	1.209	1.210	3	Nitro (+45)
RGIQTFTLPDLPY^38^DYGALEPAISGEIMQIHHQKH	36	0.907	0.908	4	Nitro (+45)

### Nitration of Tyr63 is responsible for inhibition of MSD1 activity

**Fig. 7.**
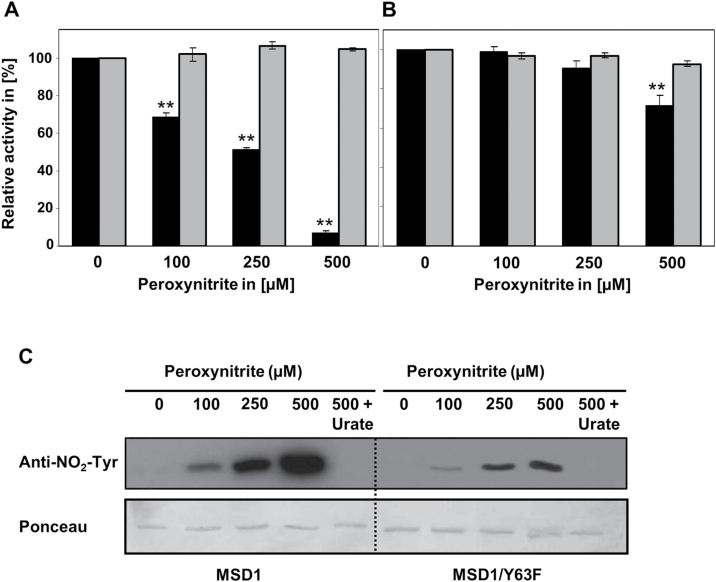
Effect of peroxynitrite on enzyme activity of MSD1/WT and MSD1/Y63F. Recombinant MSD1/WT (A) and MSD1/Y63F (B) were treated with different concentrations of peroxynitrite in the presence (grey bars) and absence (black bars) of 100 µM urate for 20min (RT, in dark). Afterwards the activity was determined. Activities without peroxynitrite were set to 100%. Values represent means±SD of three independent experiments. Asterisks (**) indicate significant differences treatment with and without urate (t-test, *P*≤0.01). Tyr nitration was detected by immunoblot analysis (C). Purified, recombinant MSD1 and MSD1/Y63 protein were separated by SDS-PAGE and blotted onto nitrocellulose membrane. Detection of nitrated Tyr residues was achieved using anti-NO_2_–Tyr antibody.

To test if nitration of Tyr63 inhibits MSD1 activity this residue was changed by site-directed mutagenesis to phenylalanine. This amino acid is structurally related to Tyr but cannot be nitrated. Wild-type and mutated MSD1 (MSD1/Y63F) were treated with different concentrations of ONOO^–^ and their activities were determined. Both wild type and modified MSD1 showed similar specific activity upon addition of decomposed ONOO^–^ (control). However, treatment with 100 and 250 µM ONOO^–^ resulted in no inhibition and 500 µM ONOO^–^ in only 30% inhibition of MSD1/Y63F, whereas wild-type MSD1 was inhibited by about 30, 50, and 90%, respectively (Fig. 7A and B). Immunoblot analyses with anti-nitrotyrosine antibodies demonstrated that overall Tyr nitration of MSD1/Y63F was much lower than that of wild-type MSD1 (Fig. 7C).

## Discussion

ROS are produced in unstressed and stressed cells as a by-product of aerobic metabolism. Plants have a well‐developed antioxidant defence, involving both limiting the formation of ROS as well as instituting their removal. SODs are enzymes that catalyse the dismutation of O_2_^–^ into oxygen and H_2_O_2_. In *Arabidopsis* seven different SODs are described, which differ in their metal-cofactor and subcellular location. Here we present MSD1, FSD3, and CSD3 as new candidates for NO-dependent post-translational regulation. GSNO, which can *S*-nitrosylate Cys residues, did not affect activity of MSD1, FSD3, and CSD3. However, incubation with the Tyr nitrating agent ONOO^–^ significantly reduced the activity of all three enzymes with MSD1 being the most sensitive isoform. Because of the variable purification efficiency of the five tested SOD isoforms we had to use different amounts of total protein. This could affect the inhibition efficiency of ONOO^–^. Therefore, we calculated the ratio of applied protein per nmol ONOO^–^ for the highest ONOO^–^ concentration used (500 µM). The highest protein amount was used in the FSD2 and MSD1 inhibition assays. As 500 µM ONOO^–^ resulted in nearly total loss of MSD1 activity this enzyme seems to be the most ONOO^–^-sensitive SOD isoform. FSD2 activity is only slightly affected by ONOO^–^ (10% with 500 µM ONOO^–^), but a stronger inhibition cannot be excluded, if lower protein amounts are used.

Similar to the plant MSD1, human and bacterial MnSODs are also very sensitive to ONOO^–^ (MacMillan-Crow *et al.*, 1998; Surmeli *et al.*, 2010). An inhibition of 30% with 100 µM ONOO^–^ might occur under physiological conditions assuming that ONOO^–^ levels in plants are similar to that in the animal system. Here the rate of ONOO^–^ production can reach 50–100 µM min^–1^ in certain cellular compartments including mitochondria (Szabo *et al.*, 2007). However, as NO production in plants is lower than in the animal system, ONOO^–^ levels might be also lower. The concentration-dependent inhibition of MSD1 positively correlated with the level of Tyr nitration (Figs 4 and 5). Inhibition of activity as well as protein nitration was prevented by the ONOO^–^ scavenger urate.

**Fig. 8.**
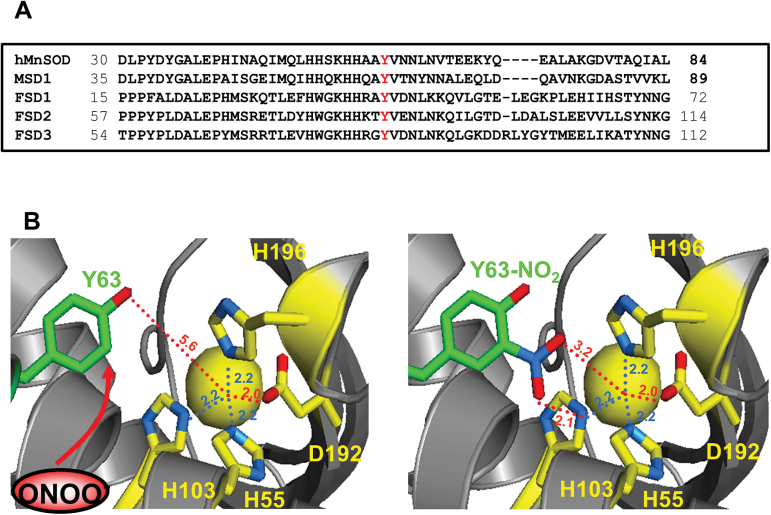
Structural illustration of nitration of conserved Tyr63 of MSD1. (A) Alignment of amino acid sequences of *Arabidopsis* FSD isoforms, MSD1, and human MnSOD (Genbank accession number: CAA32502). Dashes: Introduced gaps to maximize sequence similarity. Tyr63 of MSD1 and the corresponding Tyr in FSD1 (Tyr43), FSD2 (Tyr85), FSD3 (Tyr82), and human MnSOD (Tyr34) are highlighted in red. (B) Part of the structural model of AtMSD1 showing the substrate binding pocket. The structural model of *Arabidopsis* MSD1 was generated using SWISS-MODEL with the crystal structure of *Caenorhabditis elegans* MnSOD as template (PDB code: 3DC6). Left: the substrate binding pocket is modelled with unmodified Tyr63 (left). The position where peroxynitrite attacks the aromatic ring system of Tyr63 is indicated with a red arrow. Right: the modelled substrate binding site is shown with nitrated Tyr63. Histidine and aspartate side chains are shown in yellow; the side chain of Tyr63 is marked in green. The distance of each side chain to the manganese ion within the active site is given.

Primarily nitration of Tyr63 was responsible for the ONOO^–^ sensitivity of MSD1, as inferred by the finding that the ONOO^–^-dependent inhibition was strongly reduced in a MSD1 mutant with Tyr63 replaced by phenylalanine, which cannot be nitrated. Tyr63 is located very close to the active centre of the enzyme (5.26 Å distance) in an amino acid sequence, which is also conserved in human MnSOD (Fig. 8A). Accordingly, the corresponding Tyr34 of human MnSOD is nitrated by ONOO^–^ resulting in down-regulation of the enzymatic activity (MacMillan-Crow *et al.*, 1998; Yamakura *et al.*, 1998). It was proposed that a -NO_2_ group at ortho-position of the aromatic ring further reduces the distance to the manganese-ion in the active centre (Fig. 8B), thereby affecting access and ligation of O_2_^–^ to the substrate binding pocket. Moreover, crystal structure analyses of human MnSOD revealed a network of hydrogen bonds in the direct environment of the active centre (Perry *et al.*, 2010). Tyr34 is part of this network that probably promotes the proton transfer onto a bond O_2_^–^ anion. Nitration of the Tyr residue followed by a decrease of its pKa-value would probably deprotonate the phenol ring system causing a decrease or disruption of the hydrogen bond network. Other possible consequences of Tyr34 nitration include electrostatic interference between the nitro group and the negatively charged substrate O_2_^–^ and a shift in the redox potential of the enzyme (Edwards *et al.*, 2001). The observed inactivation of *Arabidopsis* MSD1 by ONOO^–^-mediated nitration of Tyr63 is probably based on a similar mechanism as described above for Tyr34 nitration of human MnSOD. However, it has to be mentioned that the activity of the MSD1 mutant (MSD1/Y63F) is still slightly inhibited by ONOO^–^ (Fig. 7B), suggesting that probably also nitration of other tyrosine residues affect MSD1 activity, although to a much smaller extent than nitration of Tyr63.

Previously, MnSODs of rice and potato were identified as targets for phosphorylation and oxidation, but an effect on the enzyme activity was not analysed (Bykova *et al.*, 2003; Kristensen *et al.*, 2004). It will be interesting to investigate whether Tyr nitration interferes with phosphorylation or oxidation events.

**Fig. 9.**
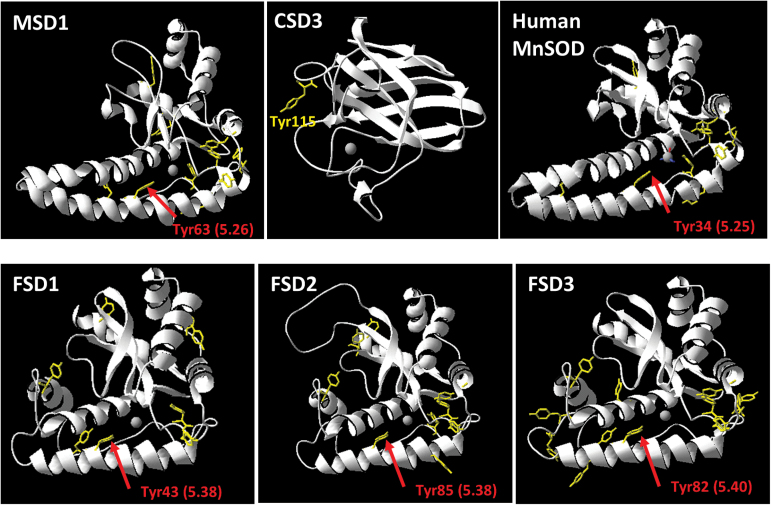
Structural model of MSD1, CSD3, human MnSOD, FSD1, FSD2, and FSD3. The structural model of *Arabidopsis* SODs was generated using SWISS-MODEL with the crystal structure of *Caenorhabditis elegans* MnSOD as template (PDBcode: PDB 3DC6). The active site ion is shown in grey. All Tyr residues are highlighted in yellow. Tyr63 of MSD1 and the corresponding tyrosine residues in FSD1 (Tyr43), FSD2 (Tyr85), FSD3 (Tyr82), and human MnSOD (Tyr34) are marked with a red arrow. The distance to the active site ion is given in brackets. Tyr115 of CSD3 is indicated in yellow.

In comparison to MnSODs much less is known about the regulation of CSDs and FSDs by ONOO^–^. *Arabidopsis* FSD3 shares 45% identity and 54% similarity in the amino acid sequence with MSD1 (Table 1). The structure is also similar between both SODs (Fig. 9). Moreover, Tyr82 of FSD3 is in the same conserved amino acid sequence as Tyr63 of MSD1 and Tyr34 of human MnSOD (Fig. 8A), all of which are located in a distance of only 5.25–5.40 Å from their active centre ion (Fig. 9). According to these sequence comparisons Tyr82 would be a good candidate regulatory site for inhibition of FSD3 by nitration. However, FSD1 and FSD2 possess the same conserved Tyr residue (Fig. 9) without being ONOO^–^ sensitive. Small variations in sequence and/or protein conformation might explain the differences in ONOO^–^ sensitivity amongst FSD isoforms as well as between FSD3 and MSD1. Alternatively, Tyr nitration of FSD3 correlates with but is not the cause of enzyme inhibition. CSDs are different from MSD1 and FSDs both in sequence as well as structure (Table 1 and Fig. 9). Amongst the three CSD isoforms of *Arabidopsis* only CSD3 has a Tyr residue. Our data demonstrate that Tyr115 is nitrated by ONOO^–^ concomitant with a reduced enzyme activity. Notably, human recombinant CuZnSOD was shown to be inhibited by tryptophan rather than Tyr nitration (Yamakura *et al.*, 2001). The exact mechanism of differential inhibition of FSD3 and CSD3 but no other FSDs and CSDs remains to be deciphered in future studies using site-directed mutagenesis and structural analyses.

Our data imply that MSD1, CSD3, and FSD3 would be partially inhibited by Tyr nitration under stress conditions, which promote the formation of ONOO^–^. Studies with *Arabidopsis* lines altered in the expression of SOD isoforms provide some hints on possible consequences of SOD inhibition. A detailed functional investigation of *Arabidopsis* FSDs revealed that chloroplastic FSD2 and FSD3 collaborate in ROS scavenging and chloroplast development (Myouga *et al.*, 2008). *fsd2*-*1 fsd3*-*1* double mutants showed an albino phenotype and were hypersensitive to oxidative stress induced by methyl viologen (Myouga *et al.*, 2008). By comparison, antisense lines of MSD1 displayed a disturbed redox homeostasis primarily in the mitochondria but to some extent also in the cytosol (Morgan *et al.*, 2008). Importantly, the mitochondrial tricarboxylic acid cycle (TCA) was interrupted through inhibition of aconitase and isocitrate dehydrogenase activity. The transgenic lines were able to adapt and did not show a decrease in downstream respiratory CO_2_ output (Morgan *et al.*, 2008). However, during short-term responses to stress, down-regulation of MSD1 might have transient but severe effects on mitochondrial TCA cycle, energy metabolism and redox homeostasis. For human kidney cells it was demonstrated that MnSOD inhibition by Tyr nitration induced irreversible oxidative injury of mitochondria during chronic rejection of human renal allografts (MacMillan-Crow *et al.*, 1996; MacMillan-Crow *et al.*, 1998).

In addition to their role in the antioxidant system SODs have relatively under-investigated functions in regulating the RNS composition and signalling. Interactions of free radicals such as O_2_^–^ and NO are important under stress conditions (Gross *et al.*, 2013). Excessive levels of O_2_^–^ during oxidative stress cause a limitation in NO bioavailability through formation of ONOO^–^. SOD in turn competes with NO for O_2_^–^ thereby preventing the formation of ONOO^–^ while favouring the accumulation of NO. Peroxiredoxin II E (PrxIIE) is another emerging player in RNS homeostasis. This hydro-peroxidase reduces peroxides to H_2_O and the corresponding alcohol using reducing equivalents from glutaredoxin or thioredoxin (Dietz, 2003). Recently it was found that PrxIIE degrades ONOO^–^ under normal growth conditions. However, after infection by an avirulent strain of *Pseudomonas syringae* PrxIIE was inhibited by *S*-nitrosylation of Cys121 resulting in ONOO^–^ accumulation and increased Tyr nitration during the hypersensitive defence response (Gaupels *et al.*, 2011*b*; Romero-Puertas *et al.*, 2007). Combining the above pieces of information would suggest that elevated levels of NO in stressed WT *Arabidopsis* cause an inhibition of PrxIIE, accumulation of ONOO^–^, and subsequently nitration-mediated inhibition of MSD1, CSD3, and FSD3. Down-regulation of the SODs would then lead to accumulation of O_2_^–^, which would further react with NO giving rise to even more ONOO^–^ in the course of a self-amplification loop. On the other side elevated levels of NO might also result in *S*-nitrosylation of NADPH oxidase (Yun *et al.*, 2011), inhibiting its activity and blunting the production of O_2_^–^. In this way the self-amplification loop would be slowed down. It is noteworthy, that MSD1, FSD3, and CSD3 are localized in mitochondria, chloroplasts, and peroxisomes, respectively, which represent major sites of ROS and NO synthesis during stress responses (Gross *et al.*, 2013). In sum, the results of our *in vitro* study provide a biochemical framework for future research aimed at deciphering how the differential regulation of SODs is involved in stress signalling, defence, or cytotoxicity.

## Supplementary data

Supplementary data are available at *JXB* online

Figure S1. Alignment of amino acid sequences of *Arabidopsis* FSD isoforms.

Figure S2. Alignment of amino acid sequences of *Arabidopsis* CSD isoforms.

Figure S3. Alignment of amino acid sequences of *Arabidopsis* FSD isoforms and MSD1.

Figure S4. Alignment of amino acid sequences of *Arabidopsis* CSD isoforms and MSD1.

Figure S5. Total SOD activity in *Arabidopsis* WT and GSNOR knock-out plants.

Figure S6. Expression analysis of *Arabidopsis* SODs.

Table S1. Oligonucleotides for cloning of superoxide dismutase nucleotide sequences and site-directed mutagenesis.

Supplementary Data
