# Supplementary material for: Differential inhibition of Arabidopsis superoxide dismutases by peroxynitrite-mediated tyrosine nitration
Source: J Exp Bot. 2014 Nov 26;66(3):989–99. doi: 10.1093/jxb/eru458 (PMC4321555; doi:10.1093/jxb/eru458)
Supplement: Supplementary Data [file supp_66_3_989__index.html]

Differential inhibition of Arabidopsis superoxide dismutases by peroxynitrite-mediated tyrosine nitration — Differential inhibition of Arabidopsis superoxide dismutases by peroxynitrite-mediated tyrosine nitration — Supplementary Data 

# Differential inhibition of *Arabidopsis* superoxide dismutases by peroxynitrite-mediated tyrosine nitration

## Supplementary Data

Data files

**Files in this Data Supplement:**

- Supplementary Data - Supplementary Data
