# Supplementary material for: Differential inhibition of Arabidopsis superoxide dismutases by peroxynitrite-mediated tyrosine nitration
Source: J Exp Bot. 2014 Nov 26;66(3):989–99. doi: 10.1093/jxb/eru458 (PMC4321555; doi:10.1093/jxb/eru458)
Supplement: Supplementary Data [file supp_eru458_jexbot133249_file001.pdf]

# Differential Inhibition of *Arabidopsis* Superoxide Dismutases by Peroxynitrite-mediated Tyrosine Nitration

**Christian Holzmeister, Frank Gaupels, Arie Geerlof, Michael Sattler, Jörg Durner, Christian Lindermayr**

**Supplemental Table S1: Oligonucleotides for cloning of superoxide dismutase nucleotide sequences and site-directed mutagenesis**

All oligonucleotides were obtained from Eurofins MWG Operon (Martinsried, Germany), diluted in water (100 µM) and stored at -20 °C.

| Name | Sequence |
|---|---|
| **Oligonucleotides for amplification of superoxid dismutase nucleotide sequences** | |
| MnSOD_ for | 5´- ATGGCGATTCGTTGTGTAGC - 3´ |
| MnSOD_rev (with stop codon) | 5´- TCAGTTGTTTTCCTTCTCATAAACC - 3´ |
| MnSOD_rev (without stop codon) | 5´- GTTGTTTTCCTTCTCATAAACCTC - 3´ |
| FeSOD1_ for | 5´- ATGGCTGCTTCAAGTGCTG - 3´ |
| FeSOD1_rev (with stop codon) | 5´- TTAAGCAGAAGCAGCCTTGG - 3´ |
| FeSOD1_rev (without stop codon) | 5´- AGCAGAAGCAGCCTTGGC - 3´ |
| FeSOD2_ for | 5´- ATGATGAATGTTGCAGTGACAG - 3´ |
| FeSOD2_rev (with stop codon) | 5´- TTAGTCAACCTCAGATACATCG - 3´ |
| FeSOD2_rev (without stop codon) | 5´- GTCAACCTCAGATACATCGAT - 3´ |
| FeSOD3_ for | 5´- ATGAGTTCTTGTGTTGTGACG - 3´ |
| FeSOD3_rev (with stop codon) | 5´- TTAAGCGATTGGGATGTTGG - 3´ |
| FeSOD3_rev (without stop codon) | 5´- AGCGATTGGGATGTTGGG - 3´ |
| CuZnSOD1_ for | 5´- ATGGCGAAAGGAGTTGCAG - 3´ |
| CuZnSOD1_rev (with stop codon) | 5´- TTAGCCCTGGAGACCAATG - 3´ |
| CuZnSOD1_rev (without stop codon) | 5´- GCCCTGGAGACCAATGAT - 3´ |
| CuZnSOD2_ for | 5´- ATGGCTGCCACCAACACAA - 3´ |
| CuZnSOD2_rev (with stop codon) | 5´- TTAGAGCGGCGTCAAGC - 3´ |
| CuZnSOD2_rev (without stop codon) | 5´- GAGCGGCGTCAAGCCA - 3´ |
| CuZnSOD3_ for | 5´- ATGGAAGCTCCTAGAGGAAATC - 3´ |
| CuZnSOD3_rev (with stop codon) | 5´- CTATAGTTTAGCATCCGCAGAT - 3´ |
| CuZnSOD3_rev (without stop codon) | 5´- TAGTTTAGCATCCGCAGATGATT - 3´ |

**<u>Oligonucleotides for GATEWAY-Cloning of superoxide dismutase nucleotide sequences</u>**

| | |
|---|---|
| attB1_MnSOD_for (with stop codon) | 5´- GGGACAAGTTTGTACAAAAAAGCAGGCTTCATG<br>GCGATTCGTTGTGTAGC - 3´ |
| attB2_MnSOD_rev (with stop codon) | 5´- GGGGACCACTTTGTACAAGAAAGCTGGGTCTCA<br>GTTGTTTTCCTTCTCATAAACC - 3´ |
| attB1_MnSOD_for (without stop codon) | 5´-GGGGACAAGTTTGTACAAAAAAGCAGGCTTCGA<br>AGGAGATAGAACCATGGCGATTCGTTGTGTAGC - 3´ |
| attB2_MnSOD_rev (without stop codon) | 5´- GGGGACCACTTTGTACAAGAAAGCTGGGTCGTTGT<br>TTTCCTTCTCATAAACCTC - 3´ |
| attB1_FeSOD1_for (with stop codon) | 5´- GGGACAAGTTTGTACAAAAAAGCAGGCTTCATGG<br>CTGCTTCAAGTGCTG - 3´ |
| attB2_FeSOD1_rev (with stop codon) | 5´- GGGGACCACTTTGTACAAGAAAGCTGGGTCTTAAG<br>CAGAAGCAGCCTTGG - 3´ |
| attB1_FeSOD1_for (without stop codon) | 5´- GGGACAAGTTTGTACAAAAAAGCAGGCTTCGAAG<br>GAGATAGAACCATGGCTGCTTCAAGTGCTG - 3´ |
| attB2_FeSOD1_rev (without stop codon) | 5´- GGGACCACTTTGTACAAGAAAGCTGGGTCAGCAG<br>AAGCAGCCTTGGC - 3´ |
| attB1_FeSOD2_for (with stop codon) | 5´- GGGGACAAGTTTGTACAAAAAAGCAGGCTTCATGA<br>TGAATGTTGCAGTGACAG - 3´ |
| attB2_FeSOD2_rev (with stop codon) | 5´- GGGGACAAGTTTGTACAAAAAAGCAGGCTTCATGA<br>TGAA - 3´ |
| attB1_FeSOD2_for (without stop codon) | 5´- GGGACAAGTTTGTACAAAAAAGCAGGCTTCGAAG<br>GAGATAGAACCATGATGAATGTTGCAGTGACAG - 3´ |
| attB2_FeSOD2_rev (without stop codon) | 5´- GGGGACCACTTTGTACAAGAAAGCTGGGTCGTCAA<br>CCTCAGATACATCGAT - 3´ |
| attB1_FeSOD3_for (with stop codon) | 5´- GGGGACAAGTTTGTACAAAAAAGCAGGCTTCATGA<br>GTTCTTGTGTTGTGACG - 3´ |
| attB2_FeSOD3_rev (with stop codon) | 5´- GGGGACCACTTTGTACAAGAAAGCTGGGTCTTAAG<br>CGATTGGGATGTTGG - 3´ |
| attB1_FeSOD3_for (without stop codon) | 5´- GGGACAAGTTTGTACAAAAAAGCAGGCTTCGAAG<br>GAGATAGAACCATGAGTTCTTGTGTTGTGACG - 3´ |
| attB2_FeSOD3_rev (without stop codon) | 5´- GGGACCACTTTGTACAAGAAAGCTGGGTCAGCGA<br>TTGGGATGTTGGG - 3´ |

| | |
|---|---|
| attB1_CuZnSOD1_for (with stop codon) | 5´- GGGACAAGTTTGTACAAAAAAGCAGGCTTCATGG CGAAAGGAGTTGCAG - 3´ |
| attB2_CuZnSOD1_rev (with stop codon) | 5´- GGGGACCACTTTGTACAAGAAAGCTGGGTCTTAGC CCTGGAGACCAATG - 3´ |
| attB1_CuZnSOD1_for (without stop codon) | 5´- GGGACAAGTTTGTACAAAAAAGCAGGCTTCGAAG GAGATAGAACCATGGCGAAAGGAGTTGCAG - 3´ |
| attB2_CuZnSOD1_rev (without stop codon) | 5´- GGGACCACTTTGTACAAGAAAGCTGGGTCGCCCTG GAGACCAATGAT - 3´ |
| attB1_CuZnSOD2_for (with stop codon) | 5´- GGGACAAGTTTGTACAAAAAAGCAGGCTTCATGG TTTCCGCGGCGAAGAAGGC - 3´ |
| attB2_CuZnSOD2_rev (with stop codon) | 5´- GGGGACCACTTTGTACAAGAAAGCTGGGTCTTAGA GCGGCGTCAAGC - 3´ |
| attB1_CuZnSOD2_for (without stop codon) | 5´- GGGACAAGTTTGTACAAAAAAGCAGGCTTCGAAG GAGATAGAACCATGGTTTCCGCGGCGAAGAAGGC - 3´ |
| attB2_CuZnSOD2_rev (without stop codon) | 5´- GGGACCACTTTGTACAAGAAAGCTGGGTCGAGCG GCGTCAAGCCA - 3´ |
| attB1_CuZnSOD3_for (with stop codon) | 5´- GGGACAAGTTTGTACAAAAAAGCAGGCTTCATGG AAGCTCCTAGAGGAAATC - 3´ |
| attB2_CuZnSOD3_rev (with stop codon) | 5´- GGGGACCACTTTGTACAAGAAAGCTGGGTCCTATA GTTTAGCATCCGCAGAT - 3´ |
| attB1_CuZnSOD3_for (without stop codon) | 5´- GGGACAAGTTTGTACAAAAAAGCAGGCTTCGAAG GAGATAGAACCATGGAAGCTCCTAGAGGAAATC - 3´ |
| attB2_CuZnSOD3_rev (without stop codon) | 5´- GGGGACCACTTTGTACAAGAAAGCTGGGTCTAGTT TAGCATCCGCAGATGATT - 3´ |

**Oligonucleotides for site-directed mutagenesis**

| | |
|---|---|
| MnSODY63F_for | 5´- CATCACCAGGCTTTTGTTACTAATTAC - 3´ |
| MnSODY63F_rev | 5´- GTAATTAGTAACAAAAGCCTGGTGATG - 3´ |

*Arabidopsis* FSDs

```
                         10        20        30        40        50
                ....|....|....|....|....|....|....|....|....|....|
FSD1-AT4G25100  ------------------------------------------MAASSAVT
FSD2-AT5G51100  MMNVAVTATPSSLLYSPLLLPSQGPNRRMQWKRNGKRRLGTKVAVSGVIT
FSD3-AT5G23310  -MSSCVVTTSCFYTISDSSIRLKSP--KLLNLSNQQRRRSLRSRGGLKVE

                         60        70        80        90       100
                ....|....|....|....|....|....|....|....|....|....|
FSD1-AT4G25100  ANYVLKPPPFALDALEPHMSKQTLEFHWGKHHRAYVDNLKKQVLGTE-LE
FSD2-AT5G51100  AGFELKPPPYPLDALEPHMSRETLDYHWGKHHKTYVENLNKQILGTD-LD
FSD3-AT5G23310  AYYGLKTPPYPLDALEPYMSRRTLEVHWGKHHRGYVDNLNKQLGKDDRLY

                        110       120       130       140       150
                ....|....|....|....|....|....|....|....|....|....|
FSD1-AT4G25100  GKPLEHIIHSTYNNGDLLPAFNNAAQAWNHEFFWESMKPGGGGKPSGELL
FSD2-AT5G51100  ALSLEEVVLLSYNKGNMLPAFNNAAQAWNHEFFWESIQPGGGGKPTGELL
FSD3-AT5G23310  GYTMEELIKATYNNGNPLPEFNNAAQVYNHDFFWESMQPGGGDTPQKGVL

                        160       170       180       190       200
                ....|....|....|....|....|....|....|....|....|....|
FSD1-AT4G25100  ALLERDFTSYEKFYEEFNAAAATQFGAGWAWLAYSN--------------
FSD2-AT5G51100  RLIERDFGSFEEFLERFKSAAASNFGSGWTWLAYKANRLDVANAVNPLPK
FSD3-AT5G23310  EQIDKDFGSFTNFREKFTNAALTQFGSGWVWLVLKR--------------

                        210       220       230       240       250
                ....|....|....|....|....|....|....|....|....|....|
FSD1-AT4G25100  ---EKLKVVKTPNAVNPLVLGSFPLLTIDVWEHAYYLDFQNRRPDYIKTF
FSD2-AT5G51100  EEDKKLVIVKTPNAVNPLVWDYSPLLTIDTWEHAYYLDFENRRAEYINTF
FSD3-AT5G23310  -EERRLEVVKTSNAINPLVWDDIPIICVDVWEHSYYLDYKNDRAKYINTF

                        260       270       280       290       300
                ....|....|....|....|....|....|....|....|....|....|
FSD1-AT4G25100  MTNLVSWEAVSARLEAAKAASA----------------------------
FSD2-AT5G51100  MEKLVSWETVSTRLESAIARAVQREQEGTETEDEENPDDEVPEVYLDSDI
FSD3-AT5G23310  LNHLVSWNAAMSRMARAEAFVNLGE----------------------PNI

                ....|.
FSD1-AT4G25100  ------
FSD2-AT5G51100  DVSEVD
FSD3-AT5G23310  PIA---
```

**Supplemental Figure S1: Alignment of amino acid sequences of *Arabidopsis* FSD isoforms.** Identical amino acids are highlighted in black, similar ones in grey. Dashes: Introduced gaps to maximize sequence similarity.

*Arabidopsis* CSDs

```
                                  10        20        30        40        50
                        ....|....|....|....|....|....|....|....|....|....|
CSD1-AT1G08830          --------------------------------------------------
CSD2-AT2G28190          MAATNTILAFSSPSRLLIPPSSNPSTLRSSFRGVSLNNNNLHRLQSVSFA
CSD3-AT5G18100          --------------------------------------------------

                                  60        70        80        90       100
                        ....|....|....|....|....|....|....|....|....|....|
CSD1-AT1G08830          -------------MAKGVAVLNSSEGVTGTIFFTQEGDGVTTVSGTVSGL
CSD2-AT2G28190          VKAPSKALTVVSAAKKAVAVLKGTSDVEGVVTLTQDDSGPTTVNVRITGL
CSD3-AT5G18100          MEAP-------RGNLRAVALIAGDNNVRGCLQFVQDISGTTHVTGKISGL

                                 110       120       130       140       150
                        ....|....|....|....|....|....|....|....|....|....|
CSD1-AT1G08830          KPGLHGFHVHALGDTTNGCMSTGPHFNPDGKTHGAPEDANRHAGDLGNIT
CSD2-AT2G28190          TPGPHGFHLHEFGDTTNGCISTGPHFNPNNMTHGAPEDECRHAGDLGNIN
CSD3-AT5G18100          SPGFHGFHIHSFGDTTNGCISTGPHFNPLNRVHGPPNEEERHAGDLGNIL

                                 160       170       180       190       200
                        ....|....|....|....|....|....|....|....|....|....|
CSD1-AT1G08830          VGDDGTATFTITDCQIPLTGPNSIVGRAVVVHADPDDLGKGGHELSLATG
CSD2-AT2G28190          ANADGVAETTIVDNQIPLTGPNSVVGRAFVVHELKDDLGKGGHELSLTTG
CSD3-AT5G18100          AGSNGVAEILIKDKHIPLSGQYSILGRAVVVHADPDDLGKGGHKLSKSTG

                                 210       220
                        ....|....|....|....|.
CSD1-AT1G08830          NAGGRVACGIIGLQG------
CSD2-AT2G28190          NAGGRLACGVIGLTPL-----
CSD3-AT5G18100          NAGSRVGCGIIGLQSSADAKL
```

**Supplemental Figure S2: Alignment of amino acid sequences of *Arabidopsis* CSD isoforms.** Identical amino acids are highlighted in black, similar ones in grey. Dashes: Introduced gaps to maximize sequence similarity.

*Arabidopsis* MSD1-FSDs

```
                          10        20        30        40        50
                  ....|....|....|....|....|....|....|....|....|....|
MSD1-AT3G10920    -MAIRCVAS---------------------RKTLAGLKETSSRLLRIRGI
FSD1-AT4G25100    ------------------------------------------MAASSAVT
FSD2-AT5G51100    MMNVAVTATPSSLLYSPLLLPSQGPNRRMQWKRNGKRRLGTKVAVSGVIT
FSD3-AT5G23310    -MSSCVVTTSCFYTISDSSIRLKSP--KLLNLSNQQRRRSLRSRGGLKVE

                          60        70        80        90       100
                  ....|....|....|....|....|....|....|....|....|....|
MSD1-AT3G10920    QTFTLPDLPYDYGALEPAISGEIMQIHHQKHHQAYVTNYNNALEQLD---
FSD1-AT4G25100    ANYVLKPPPFALDALEPHMSKQTLEFHWGKHHRAYVDNLKKQVLGTE-LE
FSD2-AT5G51100    AGFELKPPPYPLDALEPHMSRETLDYHWGKHHKTYVENLNKQILGTD-LD
FSD3-AT5G23310    AYYGLKTPPYPLDALEPYMSRRTLEVHWGKHHRGYVDNLNKQLGKDDRLY

                         110       120       130       140       150
                  ....|....|....|....|....|....|....|....|....|....|
MSD1-AT3G10920    -QAVNKGDASTVVKLQSAIKFNGGGHVN-HSIFWKNLAPSSEGGGEPPKG
FSD1-AT4G25100    GKPLEHIIHSTYNNGDLLPAFNNAAQAWNHEFFWESMKP---GGGGKPSG
FSD2-AT5G51100    ALSLEEVVLLSYNKGNMLPAFNNAAQAWNHEFFWESIQP---GGGGKPTG
FSD3-AT5G23310    GYTMEELIKATYNNGNPLPEFNNAAQVYNHDFFWESMQP---GGGDTPQK

                         160       170       180       190       200
                  ....|....|....|....|....|....|....|....|....|....|
MSD1-AT3G10920    SLGSAIDAHFGSLEGLVKKMSAEGAAVQGSGWVWLGLDK-----------
FSD1-AT4G25100    ELLALLERDFTSYEKFYEEFNAAAATQFGAGWAWLAYSN-----------
FSD2-AT5G51100    ELLRLIERDFGSFEEFLERFKSAAASNFGSGWTWLAYKANRLDVANAVNP
FSD3-AT5G23310    GVLEQIDKDFGSFTNFREKFTNAALTQFGSGWVWLVLKR-----------

                         210       220       230       240       250
                  ....|....|....|....|....|....|....|....|....|....|
MSD1-AT3G10920    -----ELKKLVVDTTANQDPLVTKGGSLVPLVGIDVWEHAYYLQYKNVRP
FSD1-AT4G25100    ------EKLKVVKTPNAVNPLVLGS---FPLLTIDVWEHAYYLDFQNRRP
FSD2-AT5G51100    LPKEEDKKLVIVKTPNAVNPLVWDY---SPLLTIDTWEHAYYLDFENRRA
FSD3-AT5G23310    ----EERRLEVVKTSNAINPLVWDD---IPIICVDVWEHSYYLDYKNDRA

                         260       270       280       290       300
                  ....|....|....|....|....|....|....|....|....|....|
MSD1-AT3G10920    EYLKN-VWKVINWKYASEVYEKENN-------------------------
FSD1-AT4G25100    DYIKTFMTNLVSWEAVSARLEAAKAASA----------------------
FSD2-AT5G51100    EYINTFMEKLVSWETVSTRLESAIARAVQREQEGTETEDEENPDDEVPEV
FSD3-AT5G23310    KYINTFLNHLVSWNAAMSRMARAEAFVNLGEPNIPIA-------------

                         310
                  ....|....|..
MSD1-AT3G10920    ------------
FSD1-AT4G25100    ------------
FSD2-AT5G51100    YLDSDIDVSEVD
FSD3-AT5G23310    ------------
```

**Supplemental Figure S3: Alignment of amino acid sequences of *Arabidopsis* FSD isoforms and MSD1.** Identical amino acids are highlighted in black, similar ones in grey. Dashes: introduced gaps to maximize sequence similarity.

*Arabidopsis* MSD1-CSDs

```
                           10        20        30        40        50
                   ....|....|....|....|....|....|....|....|....|....|
MSD1-AT3G10920     ------MAIRCVASRKTLAGLKETSSRLLRIRGIQTFTLPDLPYDYGALE
CSD1-AT1G08830     --------------------------------------------------
CSD2-AT2G28190     MAATNTILAFSSPSRLLIPPSSNPSTLRSSFRGVSLNNNNLHRLQSVSFA
CSD3-AT5G18100     --------------------------------------------------

                           60        70        80        90       100
                   ....|....|....|....|....|....|....|....|....|....|
MSD1-AT3G10920     PAISGEIMQIHHQKHHQAYVTNYNNALEQLDQAVNKGDASTVVKLQSAIK
CSD1-AT1G08830     -------------MAKGVAVLNSSEGVTGTIFFTQEGDGVTTVSGTVSGL
CSD2-AT2G28190     VKAPSKALTVVSAAKKAVAVLKGTSDVEGVVTLTQDDSGPTTVNVRITGL
CSD3-AT5G18100     -------MEAPRGNLRAVALIAGDNNVRGCLQFVQDISGTTHVTGKISGL

                          110       120       130       140       150
                   ....|....|....|....|....|....|....|....|....|....|
MSD1-AT3G10920     FNGGGHVNHSIFWKNLAPSSEGGGEPPKGSLGSAIDAHFGSLEGLVKKMS
CSD1-AT1G08830     KPG----LHGFHVHALGDTTNG---------CMSTGPHFNPDGKTHGAPE
CSD2-AT2G28190     TPG----PHGFHLHEFGDTTNG---------CISTGPHFNPNNMTHGAPE
CSD3-AT5G18100     SPG----FHGFHIHSFGDTTNG---------CISTGPHFNPLNRVHGPPN

                          160       170       180       190       200
                   ....|....|....|....|....|....|....|....|....|....|
MSD1-AT3G10920     AEGAAVQGSGWVWLGLDKELKKLVVDTTANQD-PLVTKGGSLVPLVGIDV
CSD1-AT1G08830     DANRHAGDLGNITVGDDGTATFTITDCQIPLTGPNSIVGRAVVVHADPDD
CSD2-AT2G28190     DECRHAGDLGNINANADGVAETTIVDNQIPLTGPNSVVGRAFVVHELKDD
CSD3-AT5G18100     EEERHAGDLGNILAGSNGVAEILIKDKHIPLSGQYSILGRAVVVHADPDD

                          210       220       230
                   ....|....|....|....|....|....|....|...
MSD1-AT3G10920     WEHAYYLQYKNVRPEYLKNVWKVINWKYASEVYEKENN
CSD1-AT1G08830     LGKGGHELSLATGNAGGRVACGIIGLQG----------
CSD2-AT2G28190     LGKGGHELSLTTGNAGGRLACGVIGLTPL---------
CSD3-AT5G18100     LGKGGHKLSKSTGNAGSRVGCGIIGLQSSADAKL----
```

**Supplemental Figure S4: Alignment of amino acid sequences of *Arabidopsis* CSD isoforms and MSD1.** Identical amino acids are highlighted in black, similar ones in grey. Dashes: Introduced gaps to maximize sequence similarity.

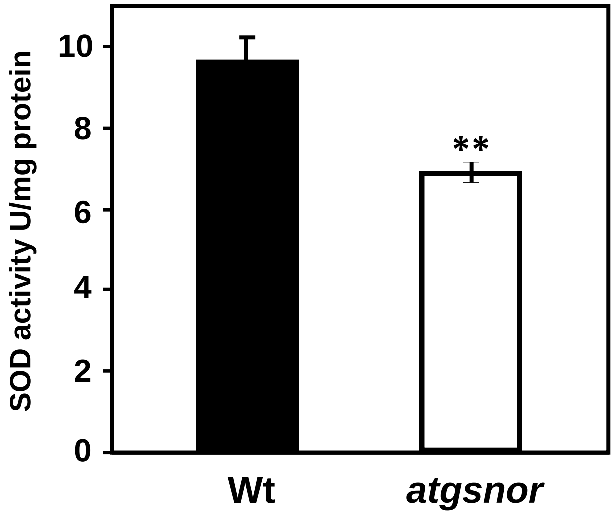


**Figure S5: Total SOD activity in *Arabidopsis* WT and GSNOR knock-out plants.** Proteins of 0,5 g leaf material of four week old plants were extracted with 1 ml extraction buffer. Total SOD activity was determined by monitoring reduction of cytochrome *c*. One unit is defined as enzyme amount necessary for 50% inhibition of cytochrome c reduction. Asterisks (**) indicate a significant difference between both lines (t-test, $p \leq 0.01$).

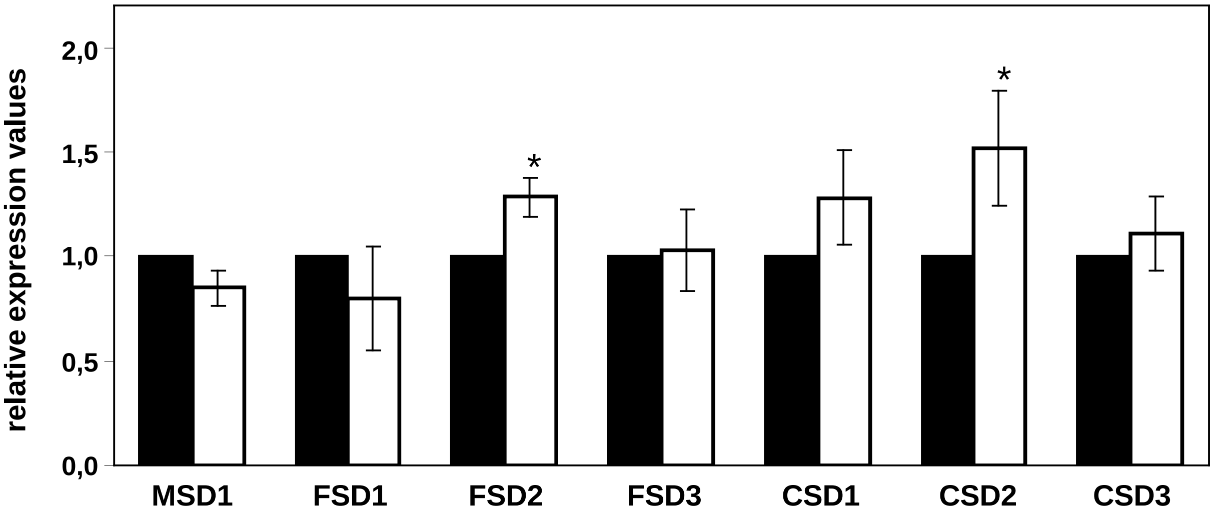


**Figure S6: Expression analysis of *Arabidopsis* SODs.**
Transcripts of *Arabidopsis* SODs were analysed by real-time quantitative PCR. Data represent the means ±SD of three experiments per lines. Normalization of real-time quantitative RT-PCR data was done by geometric averaging of multiple internal control genes (Ubiquitin 9 [At5g18380] and S16 [At5g18380 and At2g09990]).
